# Frequency Ranking of Imaging Biomarkers for Lung Cancer Risk Stratification Using a Hybrid Elastic Net Method

**DOI:** 10.3390/cancers18040582

**Published:** 2026-02-10

**Authors:** Mohamed Jaber, Emmy Stevens, Nezamoddin N. Kachouie

**Affiliations:** 1 Department of Mathematics and Systems Engineering, Florida Institute of Technology, Melbourne, FL 32901, USA; mjaber@fit.edu; 2 Department of Mechanical and Civil Engineering, Florida Institute of Technology, Melbourne, FL 32901, USA; 3 Department of Electrical Engineering and Computer Science, Florida Institute of Technology, Melbourne, FL 32901, USA

**Keywords:** lung cancer stratification, radiomic biomarkers, survival analysis, Busyness feature, SMOTE balancing, prognostic modeling, machine learning in oncology

## Abstract

Lung cancer remains the leading cause of cancer-related mortality worldwide, emphasizing the critical need for novel and robust biomarkers to improve prognostication and guide precision oncology. While traditional clinical variables such as tumor stage, age, and sex are routinely used for survival prediction, their prognostic performance is limited. Imaging biomarkers derived from radiomic analysis of advanced medical imaging have emerged as a promising class of noninvasive cancer biomarkers, enabling quantitative characterization of tumor phenotypes. In this study, we investigated the prognostic utility of radiomic imaging biomarkers, with a particular focus on the texture-based feature Busyness, and compared their performance against conventional clinical factors. Survival analyses demonstrated that Busyness achieved significantly stronger discrimination of survival outcomes than stage, age, or sex. Stratified analyses further showed that Busyness consistently remained a dominant predictor of survival across age and sex subgroups, whereas tumor stage alone provided limited prognostic separation. To address class imbalance and enhance model robustness, the Synthetic Minority Over-sampling Technique (SMOTE) was applied, further supporting the stability of the imaging biomarker findings. These results highlight the potential of radiomic imaging biomarkers as powerful prognostic tools in lung cancer and support their integration into clinical workflows. This work contributes to the growing landscape of new cancer biomarkers and provides a foundation for future studies integrating imaging biomarkers with molecular and genomic markers to achieve improved prognostic accuracy.

## Simple Summary

Accurate prediction of survival in lung cancer remains challenging, as patients with similar clinical characteristics often experience remarkably different outcomes. Traditional prognostic indicators such as tumor stage, age, and sex do not fully reflect underlying tumor aggressiveness. Medical imaging, which is already part of routine clinical care, contains additional quantitative information that can be leveraged to improve risk assessment. In this study, we demonstrate that a texture-based imaging feature, called Busyness, extracted from standard CT scans, serves as a strong indicator of survival in patients with non-small-cell lung cancer. This imaging biomarker consistently distinguishes high- and low-risk patients more effectively than conventional clinical factors across different age and sex groups. These findings suggest that imaging-derived biomarkers can enhance clinical decision-making by providing a noninvasive and objective measure of tumor behavior, thereby supporting more personalized lung cancer management.

## 1. Introduction

Lung cancer is a malignant condition characterized by the uncontrolled proliferation of abnormal cells within lung tissue, leading to tumor development that compromises surrounding healthy structures and disrupts normal physiological functions [1]. It is primarily classified into two major subtypes: non-small-cell lung cancer (NSCLC) and small-cell lung cancer (SCLC) [2]. SCLC, recognized for its aggressive and rapid growth, accounts for approximately 10–15% of lung cancer cases, whereas NSCLC, which progresses at a slower rate, represents 80–85% of diagnosed cases [2,3]. Tobacco smoking remains the leading cause of lung cancer-related deaths, responsible for nearly 81% of fatalities, underscoring the critical role of carcinogenic exposure in its pathogenesis. Lung cancer continues to be the most lethal cancer, with an estimated 340 deaths per day, 2.5 times the daily mortality rate of the second most fatal cancer type. Furthermore, it causes more annual deaths than breast, prostate, and colorectal cancers combined [4]. Notably, about 90% of lung cancer patients die within two years of diagnosis, reflecting the disease’s aggressive nature and the challenges associated with its treatment [5].

Recent advancements in lung cancer treatment have led to significant improvements in patient survival rates, particularly concerning the five-year survival metric. One notable development is the introduction of immunotherapy drugs such as pembrolizumab (Keytruda). A study led by UCLA investigators revealed that pembrolizumab treatment enabled more than 15% of individuals with advanced NSCLC to survive for at least five years, a substantial increase from the previous average five-year survival rate of 5.5%. Furthermore, 25% of patients whose tumor cells exhibited high levels of PD-L1 protein achieved five-year survival. These findings suggest that immunotherapy can significantly alter the prognosis for advanced lung cancer patients. In addition to immunotherapy, targeted therapies have emerged as effective treatments for specific lung cancer mutations. For instance, the drug Osimertinib (Tagrisso) has been approved for routine use in patients with EGFR mutation-positive NSCLC [6].

Clinical trials have demonstrated that Osimertinib, when taken with chemotherapy, can double median disease-free survival time and increase five-year survival rates. This approval marks a significant step forward in personalized cancer treatment, offering new hope to patients with this genetic profile [7]. Moreover, advancements in early detection have played a crucial role in improving survival outcomes. The implementation of mobile lung screening units, such as those initiated by the NHS in supermarket car parks, has facilitated the early diagnosis of lung cancer. These units have identified lung cancer in over 5000 individuals, with three-quarters of these cases detected at stage one or two. Early detection is vital, as patients diagnosed at an early stage are approximately 20 times more likely to survive five years or more compared to those diagnosed at later stages [8]. These advancements underscore the importance of continued research and innovation in lung cancer treatment and detection, offering renewed hope for improved patient outcomes in the future.

The high mortality rate of lung cancer has been largely attributed to its asymptomatic progression in the early stages, often leading to late detection and limited treatment options. By the time symptoms appear and a medical evaluation occurs, the disease has often advanced to a stage characterized by extensive systemic spread, significantly limiting curative treatment possibilities. Advanced-stage lung cancer, or “late-stage” disease, is marked by metastasis, a process in which epithelial tumor cells normally confined within tissues undergo an epithelial-to-mesenchymal transition (EMT), gaining migratory properties that enable them to spread and establish colonies in distant organs [9]. Lung cancer staging is a crucial factor in determining prognosis and treatment strategies, with the tumor–node–metastasis (TNM) system being the most widely used classification method [10]. This system evaluates three key aspects of tumor (T), referring to the size and extent of the primary growth; node (N), indicating the level of lymphatic involvement; and metastasis (M), assessing the spread of cancer to distant sites. Various staging classifications exist, including clinical, pathological, post-therapy, and recurrence staging. Among these, clinical staging performed before initiating treatment is particularly critical for patient stratification and serves as a focal point in treatment planning. Lung cancer is categorized into stages I through IV, with higher stages indicating more advanced disease and a poorer prognosis.

Accurate stratification of patients is essential for predicting patient outcomes [11]. Lung cancer stratification is essential for optimizing treatment decisions, predicting patient outcomes, and improving overall survival rates. Stratification involves categorizing patients based on various factors, including tumor histology, genetic mutations, staging, and biomarker expression, to tailor treatment approaches accordingly. Studies have shown that molecular profiling of lung tumors, such as identifying EGFR, ALK, and KRAS mutations, plays a crucial role in guiding targeted therapies, significantly enhancing treatment efficacy and patient prognosis [12]. Furthermore, integrating radiomic and clinical features into stratification models has been found to improve the predictive accuracy of disease progression and response to therapy [13]. By leveraging these stratification methods, clinicians can provide more personalized treatment regimens, reducing unnecessary toxicity and maximizing therapeutic benefits.

Radiomics, an evolving field in medical imaging, involves the extraction of quantitative tumor-related features from radiological scans, enabling an objective assessment of tumor morphology and heterogeneity. These high-dimensional imaging biomarkers include characteristics such as lesion shape, texture, and intensity distribution. Tumor heterogeneity, a key factor in cancer progression, has been associated with treatment resistance and an increased likelihood of metastasis. Unlike traditional biopsy-based assessments, which provide limited insights due to their reliance on discrete tissue samples, radiomics enables a comprehensive evaluation of the entire tumor, thereby improving predictive accuracy for disease severity and treatment response [14,15]. A study by Coroller et al. investigated the prognostic value of radiomic features in lung adenocarcinoma, revealing that 35 out of 635 analyzed features had significant predictive relevance for distant metastasis (DM). Additionally, 12 radiomic features, including 4 associated with DM, were linked to patient survival outcomes [16].

Artificial intelligence (AI) is playing an increasingly significant role in oncology, aiding in tumor detection, classification, and prognosis prediction. In a 2019 study, Zhou et al. developed a deep learning model to analyze magnetic resonance (MR) images of hepatic malignancies, using binary classification to differentiate between high-grade (stages I and II) and low-grade (stages III and IV) tumors, achieving an 83% classification accuracy [17]. AI applications have also been explored in cancer diagnostics as well as biomedical research applications. Nagpal et al. developed a deep learning framework to analyze histopathological slides of prostate tumors. Their model successfully identified distinct Gleason patterns, histological markers used for cancer grading and risk assessment, with accuracy comparable to expert pathologists. Notably, the deep learning algorithm achieved 97% concordance with human pathologists in cases where all experts unanimously classified the Gleason patterns. Furthermore, when tasked with overall Gleason Grade Group classification, the AI model surpassed the performance of 29 general pathologists, reaching a 70% accuracy rate compared to their 61% [18,19,20].

The research conducted here is in line with our active area of research about early detection of cancer. In our previous work, we used blood samples from healthy people with no symptoms to test DNA changes for cancer diagnosis. We investigated common CN changes among the cancer population and implemented machine learning methodologies for lung cancer clustering. Similarities and discrepancies between cancerous tissues and paired peripheral blood samples were quantified by nonlinear clustering techniques, capturing complex relationships in genomic data by analyzing DNA CNVs in lung cancer patients and using Maximal Information Coefficient (MIC). We showed that a subset of high-MIC locations could improve clustering performance, providing insights into the genomic underpinnings of lung cancer [21,22,23,24,25].

Radiomic analysis and machine learning methods such as Elastic Net and Cox regression are increasingly applied in oncologic imaging. However, the prognostic value of their comparison with established clinical factors remains insufficiently defined. In particular, it is unclear whether radiomic texture features contribute prognostic information independently or primarily alter the interpretation of variables such as stage, age, and gender. In this study, we address this gap through a survival-based evaluation of radiomic biomarkers using repeated Elastic Net ranking and hierarchical patient stratification. Rather than introducing new algorithms, we focus on clarifying how radiomic features of tumors’ texture relate to, and in some cases supersede, conventional clinical predictors, and how cancer stage becomes informative when assessed within radiomic-defined risk groups.

## 2. Data Description

The goal of this research is to develop a robust machine learning model capable of accurately predicting the cancer stage of a patient diagnosed with non-small-cell lung cancer (NSCLC). Radiomic features were extracted exclusively from the primary lung tumor regions provided in the Lung1 dataset. Tumor segmentations were pre-defined and publicly available as part of The Cancer Imaging Archive (TCIA) resource, and no additional manual or automated re-segmentation was performed in this study. All radiomic features were computed in three dimensions using voxel-based analysis. Feature extraction was performed using the PyRadiomics library, following standardized definitions consistent with the Image Biomarker Standardizations Initiative (IBSI). The dataset comprises 107 radiomic features, limited demographic information, cancer staging classifications, and survival duration. To optimize the dataset for analysis, we transformed entries representing secondary lesions into a singular feature denoting the total number of tumors present in each patient. Additionally, entries containing missing data were excluded, resulting in a final dataset comprising 398 NSCLC patients. The dataset encompasses cases classified within lung cancer stages I, II, IIIa, and IIIb, with stages IIIa and IIIb distinguished by the anatomical positioning of the tumor(s) and the distribution of affected lymph nodes. This dataset originates from a publicly accessible repository within the National Cancer Institute’s Cancer Imaging Archive. Radiomic features were systematically categorized into seven distinct groups: First-Order Statistics Features, Shape Features, Gray-Level Co-occurrence Matrix (GLCM) Features, Gray-Level Size Zone Matrix (GLSZM) Features, Gray-Level Run Length Matrix (GLRLM) Features, Neighboring Gray-Tone Difference Matrix (NGTDM) Features, and Gray-Level Dependence Matrix (GLDM) Features [26,27,28].

First-Order Statistics Features are derived from a histogram representation of the gray-level intensities within the tumor, also known as the region of interest (ROI). These features are termed “first order” because they are computed from single-pixel (2D imaging) or single-voxel (3D imaging) distributions without considering spatial relationships. Given that the dataset is based on three-dimensional imaging, the extracted radiomic features are voxel-based. Shape features quantitatively define the geometric attributes of the lesion, such as volume, surface area, sphericity, and compactness. The remaining radiomic categories are texture features, which capture the spatial heterogeneity of voxel intensities within the tumor microenvironment.

GLCM features quantify the spatial relationships between pairs of voxels with specific gray-level values, providing insight into local textural patterns within the ROI. GLSZM features assess the number of zones formed by contiguous voxels sharing identical intensity values, allowing for an evaluation of tumor homogeneity. GLRLM features characterize the distribution and length of consecutive voxel sequences showing uniform intensity levels, which can be indicative of tissue irregularities. NGTDM features analyze the contrast between individual voxel intensities and the mean intensity of surrounding voxels within a specified neighborhood, thereby capturing local texture variations. Finally, GLDM features measure the degree of dependence between voxel groups of a given size and a central reference voxel, providing information about the complexity and uniformity of the tumor texture. Radiomic Busyness reflects rapid gray-level changes between the central pixel or voxel and its neighbors. It means a high spatial frequency of intensity changes, such that a ROI comprising many small areas with noticeably different gray levels will have greater Busyness values. Table 1 summarizes the baseline demographic and clinical characteristics of the NSCLC cohort to provide a clinical context for the subsequent survival and stratification analyses [15,26,29].

The Lung1 dataset was selected for this study because it provides a well-curated, clinically relevant foundation for radiomics-based prognostic analysis in non-small-cell lung cancer. It includes a clearly defined NSCLC cohort with standardized tumor segmentations, harmonized radiomic feature extraction, and long-term survival follow-up, all of which are essential for robust survival modeling. The availability of expert-defined tumor contours minimizes variability related to segmentation. It ensures consistency across patients, allowing the analysis to focus on the prognostic contribution of imaging features rather than contouring differences.

Importantly, Lung1 has been widely adopted in prior radiomics studies, making it a de facto benchmark dataset and enabling direct methodological comparison with the existing literature. Although derived from a single public cohort, the dataset captures substantial clinical heterogeneity across tumor stages, patient demographics, and survival outcomes, reflecting the realistic presentations of NSCLC encountered in routine practice. This balance between standardization and variability supports its suitability for evaluating the prognostic value of radiomic biomarkers, particularly texture-based features such as Busyness, while also acknowledging the need for future multi-institutional validation to assess generalizability further.

## 3. Methods

**Figure 1:**
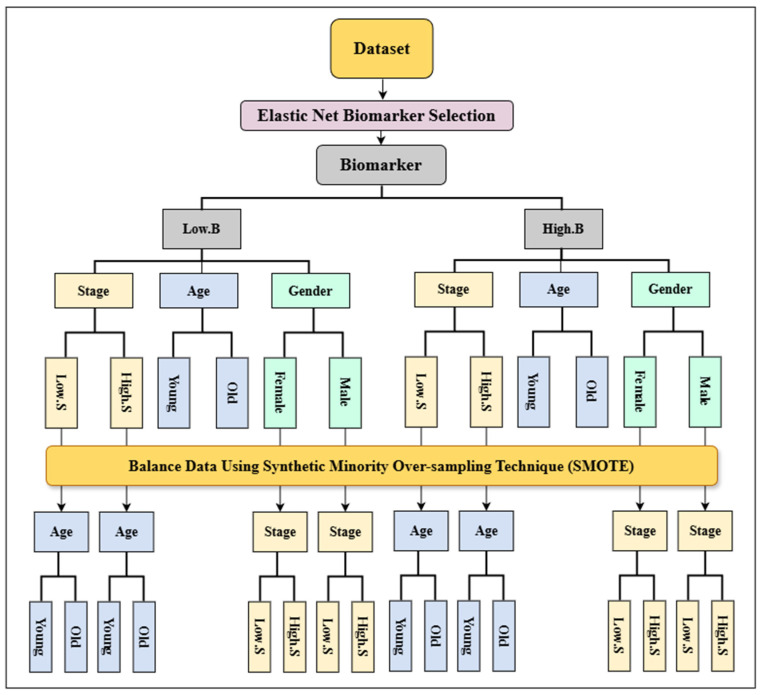
Schematic of the proposed lung cancer stratification approach.

The flow diagram of the proposed Elastic Net Biomarker selection and multimodal data integration is depicted in Figure 1. The methodological framework employed in this study follows a branching, multi-step process comprising 12 distinct procedures. Initially, the impactful biomarker(s) in predicting patient survival were identified using Elastic Net. Then, the most predictive biomarker (Busyness) was employed to stratify the data into two classes (Low.B vs. High.B) based on biomarker median value. Following this, stage, age, and gender were integrated one at a time to further branch out and stratify the patients into subgroups. At this point, the issue of class imbalance was addressed to be able to branch out and further stratify the patients into more distinctive subgroups. The Synthetic Minority Over-Sampling Technique (SMOTE) was applied, after which the dataset was stratified into subgroups based on Biomarker, Stage, Gender, and Age. The proposed methodology is explained in detail below.

### 3.1. Binary Data Mapping

For further investigation of patients’ survival, patients were stratified into different groups based on clinical factor(s) such as cancer stage, demographics such as age (below or above the median), and gender, and the quantified value of a biomarker (such as Busyness). This procedure is explained below.

#### 3.1.1. Low and High Lung Cancer Stages

Based on the overall cancer stage, patients were divided into four groups, including I, II, IIIa, and IIIb. First, patients were binned into binary classes, Low Stage (277 patients) and High Stage (121 patients), where stages I and II were combined in the Low-Stage class, and stages IIIa and IIIb were combined into the High-Stage class.

#### 3.1.2. Young and Old Lung Cancer Patients

Based on the median age, patients were divided into two groups: Young (199 patients) and Old (199 patients). First, the median age of the dataset was calculated, and the patients were then split into two classes based on whether their age was below or above the median. The group of patients with ages below the median was classified as the “Young” group, and those with ages above the median were classified as the “Old” group.

#### 3.1.3. Female and Male Lung Cancer Patients

Based on gender, patients were divided into two groups: Male (273 patients) and Female (125 patients). The data were split into two classes based on the gender of the patients, with males classified as the “Male” group and females classified as the “Female” group.

#### 3.1.4. Low and High Biomarker Value

Based on the median biomarker value, patients were divided into two groups: Low and High Biomarker. The median biomarker value were calculated, and patients were then split into two classes based on whether their biomarker value were below or above the median. The group of patients with biomarker values below the median were classified as the “Low Biomarker” group (199 patients), and those with biomarker values above the median were classified as the “High Biomarker” group (199 patients) for Busyness as a biomarker.

### 3.2. Addressing Class Imbalance

**Figure 2:**
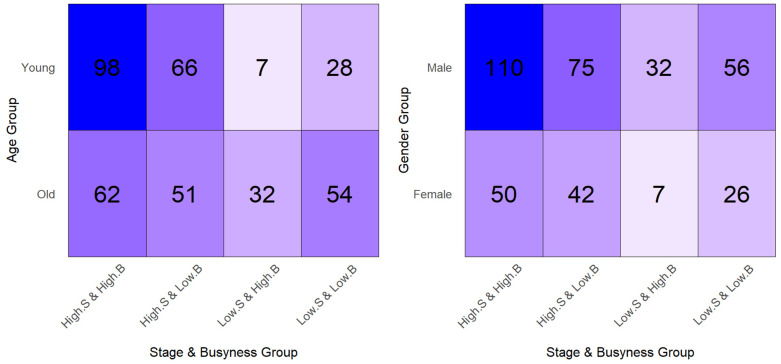
Patient count heatmap. Age group (**left**) and Gender group (**right**) in association with Stage (Low and High) and biomarker Busyness (Low and High).

The High-Stage group (277 out of 398 patients), which was created by combining stages IIIa and IIIb, has a higher occurrence than the Low-Stage group. It means the size of the Low-Stage class is approximately one-third of the High Stage in binary classification. As the dominant class may skew the predictive power of the model when the dataset is imbalanced. Figure 2 demonstrates a heatmap illustrating the patient counts across age groups (Young, Old) and gender groups (Female, Male) in relation to Stage (High, Low) and biomarker Busyness (High, Low). Note that the color intensity indicates the patient count for each combination of these categories. Significant differences in the number of patients across the different combinations may lead to inaccurate conclusions. For instance, the group “Young, Low.S, Low.B” consists of only 7 patients out of 398 patients (3 females, 4 males), which is a substantial imbalance. Such narrow sample sizes can limit the effectiveness of the Cox regression model and hinder the ability to observe clear survival time separation between groups. To address this issue, the Synthetic Minority Over-sampling Technique (SMOTE) was utilized to generate synthetic samples for underrepresented groups. To mitigate extreme class imbalance in downstream stratified subgroup analyses, SMOTE was applied solely for explanatory and comparative purposes. Importantly, all primary survival analyses and conclusions regarding prognostic performance were derived from the original, non-oversampled dataset. SMOTE-based analyses were used only to assess the stability of observed stratification patterns under balanced subgroup conditions and were not employed to estimate hazard ratios or infer absolute survival probabilities.

### 3.3. Biomarker Selection

To identify the most predictive biomarkers for survival outcomes, we utilized Elastic Net regularization within the Cox Proportional Hazards (Cox PH) framework described below.

#### 3.3.1. Elastic Net Feature Selection

Elastic Net is a machine learning method that has recently emerged and found many applications in medical research, public health, and the economy [30,31,32,33]. This method combines the strengths of both Lasso (L1) and Ridge (L2) regularization techniques. Elastic Net regularization can improve the feature selection to avoid overfitting by addressing multicollinearity among a large number of predictors, which is a common problem in survival data. Elastic Net regularization enhances the Cox proportional hazards (Cox PH) model by imposing both L1 and L2 penalties on the regression coefficients. The L1 regularization (Lasso) enforces sparsity in the model by setting irrelevant or redundant biomarker coefficients to zero, effectively performing feature selection. In contrast, the L2 regularization (Ridge) shrinks the coefficients of correlated biomarkers toward zero, thus mitigating multicollinearity, which is common in datasets with highly correlated features. This dual regularization approach helps to retain predictive biomarkers while ensuring stability in coefficient estimates.

The proposed approach, integrating Elastic Net regularization within the Cox PH model, allowed for the development of a parsimonious and interpretable model, which is crucial for prognostic assessment in clinical settings. The final model was evaluated based on its predictive accuracy and stability, providing a robust tool for survival prediction and identifying potential biomarkers that could guide personalized treatment strategies.

#### 3.3.2. Biomarker Frequency Ranking

The mixing parameter α (ranging from 0 to 1) in Elastic Net controls the balance between the Lasso and Ridge effects. Feature reduction was implemented using a set of 11 values with intervals of 0.1 for α ranging from 0 (pure Ridge regularization) to 1 (pure Lasso regularization). For each value of α, an Elastic Net-penalized Cox Proportional Hazards model was fitted. The regularization parameter λ was selected using 10-fold cross-validation, and the value minimizing the cross-validated partial likelihood deviance was used. This procedure was repeated using Monte Carlo resampling. At each α−λ setting, radiomic features with non-zero coefficients were recorded. Feature importance was summarized by counting the frequency with which each feature was selected across all runs. A total of 1100 Elastic Net models were evaluated (100 runs × 11 α values). Among the 107 radiomic features, Busyness was selected most frequently. This frequency-based strategy emphasizes biomarker stability and reproducibility rather than reliance on a single optimized model, which is particularly important in high-dimensional radiomic survival analysis. By aggregating feature selection frequencies across repeated cross-validation runs and multiple regularization settings, this approach reduces dependence on any single data split or cross-validation fold.

It is worth mentioning that Busyness belongs to the NGTDM (Neighborhood Gray-Tone Difference Matrix) radiomics category, which contains other key biomarkers, such as Strength, which are consistently selected as significant features among the top-ranked biomarkers. Meanwhile, biomarkers like Maximum 2D Diameter Slice, which belong to the “Shape/Morphological Features” category, and Size Zone Non-Uniformity, which belongs to the “Gray-Level Size Zone Matrix (GLSZM)” category, were also often among the top-ranked biomarkers. The list of top 16 biomarkers, along with their associated frequencies and radiomics categories, is shown in Table 2. Top-ranked biomarkers span several categories, including Shape/Morphological Features, Neighborhood Gray-Tone Difference Matrix (NGTDM)**,** and the Gray-Level Difference Method (GLDM). As shown in Table 2, among the top five biomarkers, two belong to the NGTDM Texture features, two belong to the Shape features, and one belongs to the GLSZM Texture features. The proposed ranking methodology indicates that Busyness is highly relevant in capturing key survival characteristics, especially under moderate to high regularization strengths.

**Figure 3:**
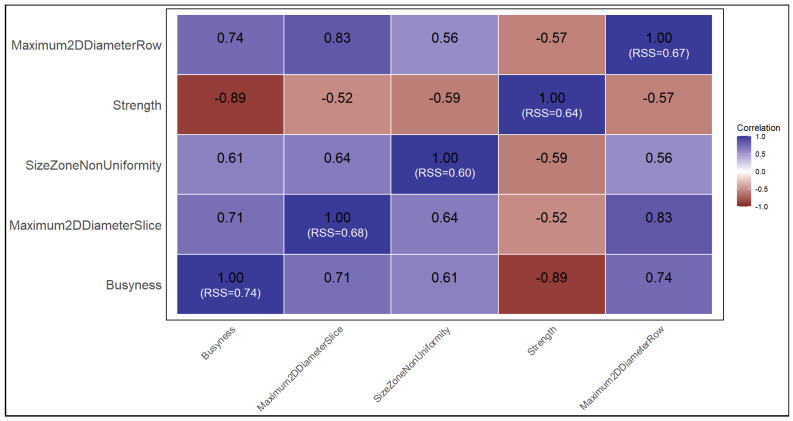
Correlation heatmap of the top-five ranked radiomic features with same Elastic Net frequency.

Although several radiomic features exhibited identical high frequency ranks, correlation analysis revealed strong pairwise associations among the top-ranked biomarkers (|ρ| ≥ 0.6), indicating substantial redundancy across feature categories (Figure 3). Specifically, Busyness demonstrated consistently strong correlations with texture-based features such as Strength and Size Zone Non-Uniformity, as well as shape-related metrics including Maximum 2D Diameter Slice and Maximum 2D Diameter Row. This central correlation pattern is quantitatively reflected by Busyness, exhibiting the highest representativeness strength score (RSS = 0.74), defined as the mean absolute Spearman correlation with the remaining top-ranked features. The elevated RSS indicates that Busyness captures overlapping information shared across multiple radiomic families. To reduce multicollinearity while preserving prognostic information, Busyness was therefore selected as a representative feature for downstream survival stratification analyses.

## 4. Results

**Figure 4:**
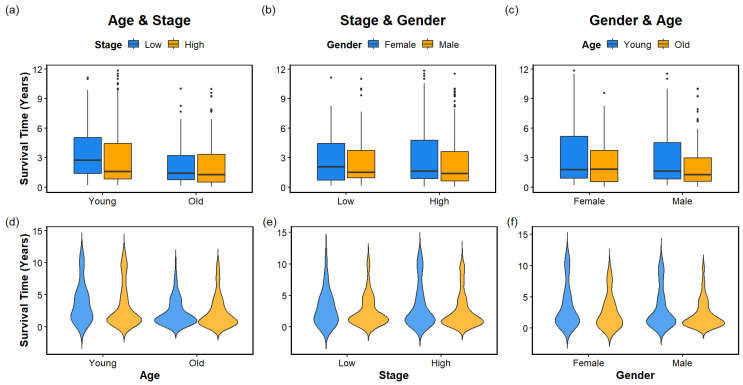
Box and violin (distribution) plots of survival time in association with demographic and clinical factors. Box plots are shown in the top row and violin plots in the bottom row. The left column shows Age and Stage (**a**,**d**), the middle column shows Stage and Gender (**b**,**e**), and the right column shows Gender and Age (**c**,**f**).

Figure 4 shows box plots (top row) and violin plots (bottom row) depicting survival time distributions across demographic and clinical categories.

### 4.1. Box Plots (Top Row)

Figure 4 (top row) presents box plots illustrating the distribution of survival time across combinations of age, tumor stage, and gender.

In the age–stage comparison (top left), survival time is shown for younger and older patients (stratified by the median age of 65 years) across low- and high-stage disease. Both age groups exhibit broadly similar survival distributions within each stage category. However, high-stage patients tend to show slightly lower median survival times compared with low-stage patients. The presence of outliers in all groups indicates substantial inter-patient variability in survival outcomes.

The stage–gender comparison (top middle) displays survival time across low- and high-stage disease, stratified by sex. Survival distributions appear broadly comparable between males and females within each stage category, although modest differences in median survival time are observed. Outliers are present across all subgroups, reflecting heterogeneity in survival even among patients with similar stage and sex.

The gender–age comparison (top right) illustrates survival time for male and female patients further stratified by age group. Median survival times are generally comparable between males and females, while older patients tend to demonstrate slightly lower survival across both sexes. The substantial overlap in survival distributions suggests that age alone does not produce strong separation in survival outcomes when considered independently.

### 4.2. Violin Plots (Bottom Row)

Figure 4 (bottom row) presents violin plots depicting the distribution and density of survival time across combinations of age, tumor stage, and gender.

In the age–stage comparison (bottom left), the violin plots illustrate smoothed density estimates of survival time for younger and older patients across low- and high-stage disease. High-stage patients demonstrate shorter survival times with more compact distributions, whereas low-stage patients exhibit broader survival distributions, indicating greater variability. Higher density at lower survival times is observed among high-stage patients, consistent with poorer survival outcomes.

The stage–gender comparison (bottom middle) shows survival time distributions for male and female patients within each stage category. Low-stage disease is associated with wider survival distributions for both sexes, while high-stage disease exhibits more concentrated distributions toward shorter survival durations. The overall shape of the violin plots suggests largely comparable survival patterns between males and females across stages, with males showing slightly greater variability.

The gender–age comparison (bottom right) displays survival distributions for male and female patients stratified by age group. Younger patients (≤65 years) show broader distributions, reflecting increased variability in survival outcomes, whereas older patients (>65 years) generally demonstrate shorter survival times. Differences between male and female distributions remain modest, indicating that gender alone does not produce strong separation in survival outcomes. Collectively, these descriptive analyses suggest that conventional clinical variables alone provide limited separation of survival distributions, motivating subsequent time-to-event survival analyses to formally evaluate their prognostic value.

### 4.3. Survival Analysis of Clinical Variables and Radiomic Biomarkers

**Figure 5:**
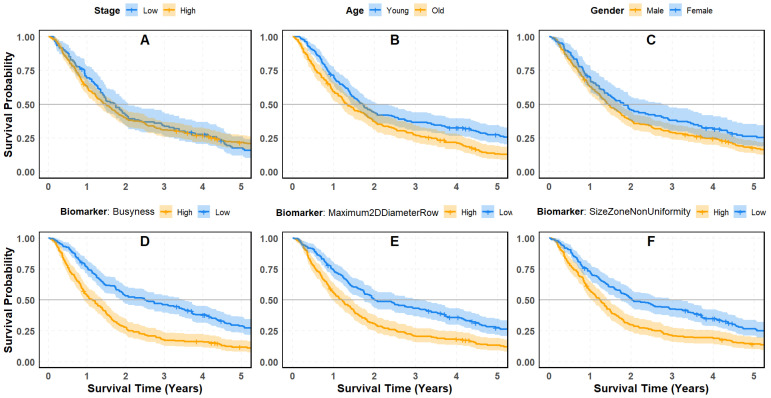
Kaplan–Meier survival curves illustrating patient risk groups based on clinical, demographic, and imaging biomarkers: (**A**) cancer stage (Low vs. High); (**B**) age group (Young vs. Old); (**C**) gender (Female vs. Male); (**D**) biomarker Busyness (Low vs. High); (**E**) biomarker Maximum 2D Diameter Row (Low vs. High); (**F**) biomarker Size Zone Non-Uniformity (Low vs. High).

Figure 5A–C, illustrate lung cancer patient survival over a five-year period based on stage, age, and gender, respectively.

In Panel (A), patients are categorized into two groups based on cancer Stage: “Low” (blue) and “High” (orange). Initially, patients in the High-Stage group exhibit slightly lower survival probabilities. However, as time progresses, the survival difference between the two groups diminishes, with their confidence intervals overlapping significantly. The *p*-value (0.53) suggests no statistically significant difference in survival outcomes based on stage. This finding implies that, in its current classification, stage alone may not serve as a strong predictor of long-term survival. Panel (B) compares survival probabilities between “Younger” and “Older” patients. Throughout the five-year period, younger patients consistently demonstrate higher survival rates. While some overlap exists in the confidence intervals, the survival curves show some separation compared to the stage-based analysis. The *p*-value (0.00039) confirms a statistically significant difference, indicating that age is a more reliable predictor of survival than stage. Panel (C) examines survival differences between male and female patients. The results show that females tend to have higher survival probabilities than males, though the confidence intervals exhibit moderate overlap. The *p*-value (0.025) suggests a statistically significant but less pronounced difference compared to age. This indicates that while gender is a better predictor of survival than stage, it is not as strong a determinant as age.

### 4.4. Radiomic Biomarkers as Predictors of Survival

In contrast to conventional clinical factors, survival analysis of the top three radiomic biomarkers: Busyness, Maximum 2D Diameter Row, and Size Zone Non-Uniformity, reveals a significantly stronger separation in survival probabilities (Figure 5D–F). These findings suggest that radiomic features provide superior predictive value for lung cancer prognosis. Panel (D) evaluates the radiomic biomarker Busyness, which quantifies the frequency of rapid intensity variations in medical images and serves as an indicator of texture complexity. Patients with High-Busyness values (orange) exhibit significantly lower survival probabilities compared to those with Low Busyness values (blue). The survival curves are well-separated with marginal overlap of confidence intervals, and the *p*-value (<0.0001) confirms a highly significant difference. Busyness consistently ranks as the top-performing biomarker in Elastic Net simulations, reinforcing its reliability and predictive power. Panel (E) assesses the Maximum 2D Diameter Row, a shape-based radiomic feature representing the widest cross-sectional tumor size. Patients with larger tumor diameters experience markedly lower survival probabilities, as evidenced by the clear divergence of survival curves and low overlap in confidence intervals. The *p*-value (<0.0001) further validates the statistical significance of this feature, underscoring its importance in prognosis. As a key morphological characteristic, the Maximum 2D Diameter Row repeatedly ranks among the top predictive features across different regularization strengths. Panel (F) examines Size Zone Non-Uniformity, a texture feature derived from the Gray-Level Size Zone Matrix (GLSZM). Patients with higher non-uniformity values exhibit significantly lower survival probabilities compared to those with more uniform size zones. The survival curves are well-separated, and the *p*-value (0.00022) confirms the statistical significance of this feature in predicting patient outcomes.

### 4.5. Implications for Survival Stratification

**Figure 6:**
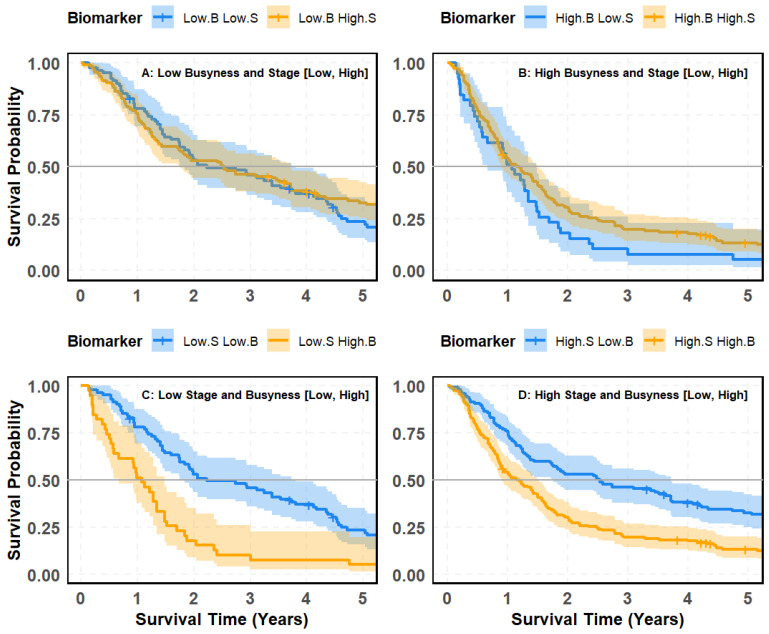
Kaplan–Meier survival curves comparing patient risk groups based on imaging biomarker Busyness and clinical stage: (**A**) low Busyness stratified by stage (Low vs. High); (**B**) high Busyness stratified by stage (Low vs. High); (**C**) low stage stratified by Busyness (Low vs. High); (**D**) high stage stratified by Busyness (Low vs. High).

The results highlight the superior predictive capability of radiomic biomarkers, particularly Busyness, Maximum 2D Diameter Row, and Size Zone Non-Uniformity, compared to conventional clinical variables such as stage, age, and gender. These findings support the integration of quantitative imaging features into lung cancer prognosis models to enhance survival stratification. Given the strong predictive performance of radiomic biomarkers, this study will focus on Busyness, the best-performing biomarker identified by Elastic Net regularization and the Cox Proportional Hazards model. Further analysis will explore the prognostic value of Busyness across different age and gender groups. Additionally, we will compare models incorporating stage, age, and gender with those integrating radiomic features to determine whether the inclusion of imaging biomarkers leads to improved survival prediction for patient stratification. As depicted in Figure 6, a comparative analysis of the survival plots further underscores the superior prognostic value of radiomic biomarkers, particularly Busyness, over traditional clinical factors such as stage.

#### 4.5.1. Stage and Busyness Combinations: Limited Predictive Value

Figure 6A,B assess the impact of combining stage (S) with Busyness (B) on survival prediction. While some differences in survival trends are observed, substantial overlap exists between groups.

Panel (A) compares survival outcomes between patients with Low Busyness, stratified by Low and High Stage. The resulting *p*-value (0.19) indicates no statistically significant difference, suggesting that stage does not contribute meaningfully to survival prediction when paired with Busyness. Panel (B) examines High-Busyness patients, again stratified by stage. While the survival curves exhibit slightly more separation, the *p*-value (0.06) remains above the conventional significance threshold. This reinforces the notion that stage, when used in conjunction with Busyness, does not substantially enhance predictive accuracy.

#### 4.5.2. Busyness as a Stronger Predictor When Stratified by Stage

In contrast, Figure 6C,D show a much clearer separation in survival probabilities when patients are stratified by stage within Busyness groups. Panel (C) focuses on Low-Stage patients, revealing that individuals with High-Busyness experience significantly worse survival outcomes than those with Low Busyness. The survival curves are well-separated, with a highly significant *p*-value (<0.0001) confirming the strong predictive power of Busyness. Panel (D) presents similar findings for High-Stage patients, where those with High Busyness again exhibit markedly poorer survival outcomes compared to their Low Busyness counterparts. The *p*-value (<0.0001) further reinforces the statistical significance of this distinction. Taken together, these results highlight an asymmetric relationship between tumor stage and Busyness in survival stratification. As shown in Panels (A) and (B), when the stage is evaluated within Busyness-defined subgroups, survival separation remains weak and inconsistent, indicating limited added prognostic value from stage once radiomic heterogeneity is accounted for. In contrast, as illustrated in Panels (C) and (D), when Busyness is evaluated within stage-defined cohorts, survival curves separate clearly and reproducibly across both low- and high-stage disease. This pattern indicates that Busyness captures prognostic information not adequately reflected by conventional staging and suggests that the stage becomes clinically informative primarily after accounting for radiomic texture heterogeneity. Overall, these findings support the broader conclusion that radiomic biomarkers, particularly texture-based features such as Busyness, provide stronger survival discrimination than traditional clinical parameters and may enhance patient stratification when integrated into prognostic models.

### 4.6. Interaction Between Busyness and Age in Survival Prediction

**Figure 7:**
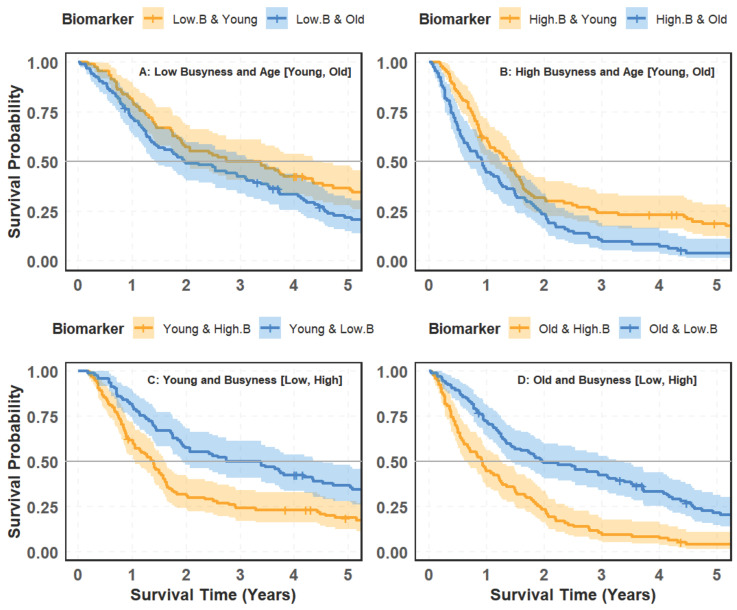
Kaplan–Meier survival curves illustrating patient risk groups based on the interaction between the Busyness imaging biomarker and age: (**A**) low Busyness stratified by age (Young vs. Old); (**B**) high Busyness stratified by age (Young vs. Old); (**C**) young patients stratified by Busyness (Low vs. High); (**D**) old patients stratified by Busyness (Low vs. High).

Figure 7 analyzes the interplay between the top biomarker, Busyness (B), and age groups (young vs. old) in predicting survival outcomes.

#### 4.6.1. Survival Stratified by Age Within Busyness Levels

Figure 7A,B investigate survival differences between young and old patients within Low and High-Busyness groups, respectively. Panel (A) focuses on patients with Low Busyness, where younger individuals exhibit higher survival probabilities than older individuals. However, the difference is moderate, with a *p*-value of 0.028, indicating statistical significance but a limited effect size. Panel (B) examines patients with High Busyness, revealing a more pronounced survival disparity between age groups. Young patients still demonstrate better survival outcomes, with a stronger statistical significance (*p*-value = 0.0037). This reinforces the well-established survival advantage of younger individuals, even when controlling for radiomic biomarker-defined subgroups.

#### 4.6.2. Survival Stratified by Busyness Within Age Groups

**Figure 8:**
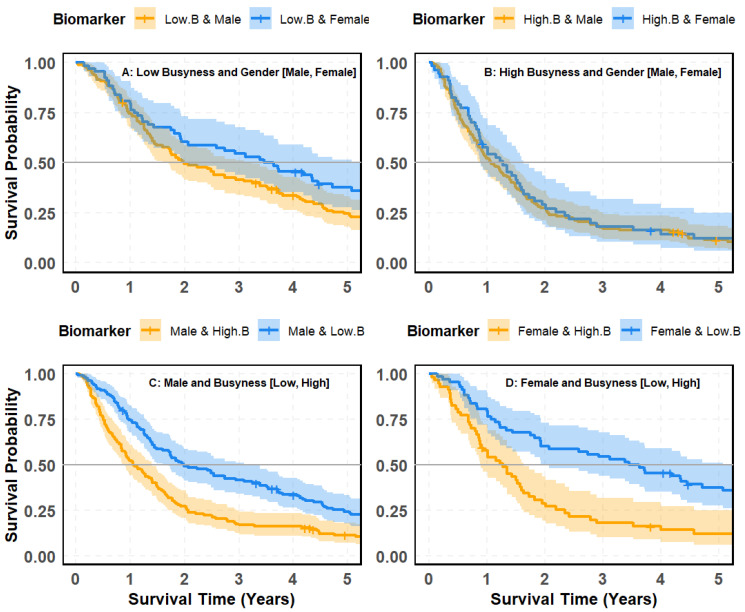
Kaplan–Meier survival curves illustrating patient risk groups based on the interaction between the Busyness imaging biomarker and gender: (**A**) low Busyness stratified by gender (Male vs. Female); (**B**) high Busyness stratified by gender (Male vs. Female); (**C**) male patients stratified by Busyness (Low vs. High); (**D**) female patients stratified by Busyness (Low vs. High).

A more distinct pattern emerges in Figure 7C,D, where patients are grouped by Busyness level within each age category. Panel (C) examines survival differences within the young population, showing that individuals with High-Busyness experience significantly worse outcomes than those with Low Busyness. The survival curves display clear separation, with a *p*-value of 0.0015, confirming strong statistical significance. Panel (D) highlights an even sharper distinction among older patients, where those with High-Busyness face substantially lower survival probabilities compared to those with Low Busyness. The *p*-value (<0.0001) indicates an extremely strong association, emphasizing the prognostic value of Busyness in this age group. Figure 8 explores the relationship between the top radiomic biomarker, Busyness (B), and gender (male vs. female) in predicting survival outcomes.

Taken together, these results demonstrate a clear asymmetry in the interaction between age and Busyness for survival stratification. As shown in Panels (A) and (B), when age is evaluated within Busyness-defined subgroups, survival differences between younger and older patients are present but remain moderate, even within the high-Busyness group, where the disparity is more pronounced. In contrast, Panels (C) and (D) show substantially stronger and more consistent survival separation when Busyness is evaluated within age-defined cohorts. In both young and older patients, High Busyness is associated with markedly poorer survival outcomes, with stronger statistical significance than that observed for age-based comparisons. This pattern indicates that while age retains prognostic relevance, Busyness provides superior discriminatory power across age groups and captures survival-related information that extends beyond age alone. Overall, these findings support the role of Busyness as a dominant stratifying factor for survival prediction, with age exerting a secondary modifying effect.

### 4.7. Interaction Between Busyness and Gender in Survival Prediction

Figure 8 investigates the interaction between the radiomic texture biomarker Busyness (B) and gender (female vs. male) in stratifying survival outcomes. By examining survival patterns across Busyness-defined subgroups and, conversely, Busyness effects within gender-defined cohorts, this analysis evaluates whether gender provides independent prognostic value or whether survival differences are predominantly driven by radiomic heterogeneity captured by Busyness.

#### 4.7.1. Survival Stratified by Gender Within Busyness Levels

Figure 8A,B evaluate whether gender influences survival outcomes within Low- and High-Busyness groups, respectively. Panel (A) compares survival between males and females with Low Busyness. While females exhibit slightly better survival outcomes, the difference is minimal, with a *p*-value of 0.87, indicating no statistically significant distinction. Panel (B) analyzes males and females with High Busyness, revealing a similar trend where females again show modestly better survival rates. However, the separation remains weak, with a *p*-value of 0.4, confirming no significant difference. These findings suggest that when using gender alone within Busyness-defined groups, survival differentiation remains statistically insignificant, highlighting gender’s limited predictive value.

#### 4.7.2. Survival Stratified by Busyness Within Gender Groups

A much stronger survival separation emerges when stratified by Busyness level within gender groups (Figure 8C,D). Panel (C) examines survival among female patients, showing that those with High Busyness experience substantially worse survival outcomes compared to those with Low Busyness. The survival curves display clear separation, supported by a statistically significant *p*-value of 0.001. Panel (D) presents survival differences among male patients, where High Busyness is associated with dramatically lower survival probabilities than Low Busyness. The separation is even more pronounced, with a highly significant *p*-value (<0.0001). Taken together, these findings reveal a clear asymmetry in the prognostic roles of Busyness and gender in survival stratification. When gender is evaluated within Busyness-defined subgroups, survival separation remains weak and statistically insignificant, indicating that sex alone contributes limited independent prognostic information once radiomic heterogeneity is considered. In contrast, when Busyness is evaluated within gender-defined cohorts, survival curves separate sharply and consistently for both male and female patients, with highly significant differences observed across Busyness levels. This pattern demonstrates that Busyness captures dominant prognostic information that is not adequately reflected by gender and reinforces radiomic texture heterogeneity as a primary driver of survival outcomes. These results further support the use of Busyness as a robust stratifying biomarker, with demographic variables such as gender serving a secondary, context-dependent role in survival prediction.

### 4.8. SMOTE-Balanced Survival Stratification: Busyness–Stage Interaction Across Age Subgroups

**Figure 9:**
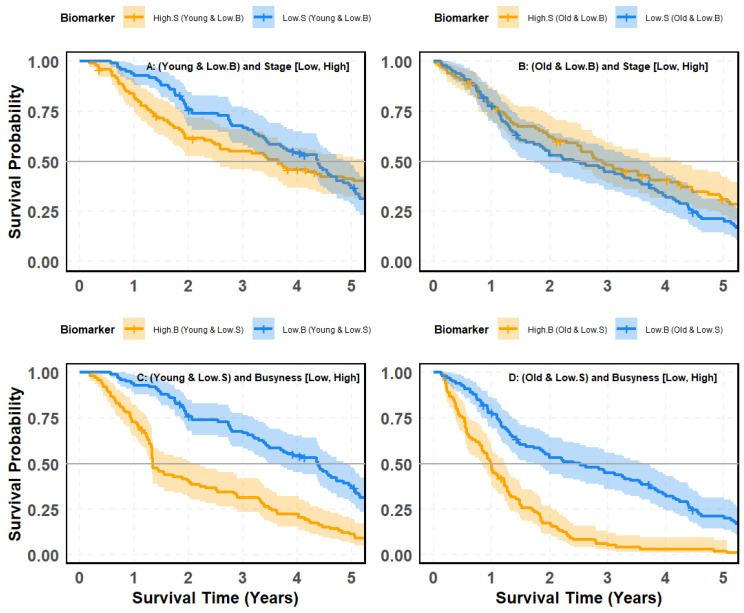
Kaplan–Meier survival curves based on SMOTE-balanced data illustrating patient risk groups from the interaction between the Busyness imaging biomarker and age: (**A**) young patients with low Busyness stratified by stage (Low vs. High); (**B**) old patients with low Busyness stratified by stage (Low vs. High); (**C**) young patients with low stage stratified by Busyness (Low vs. High); (**D**) old patients with low stage stratified by Busyness (Low vs. High).

**Figure 10:**
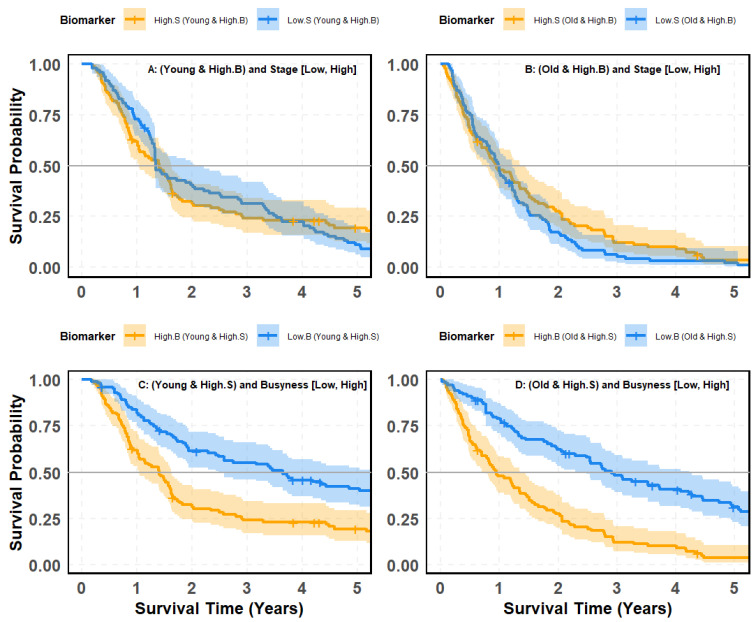
Kaplan–Meier survival curves based on SMOTE-balanced data illustrating patient risk groups from the interaction between the Busyness imaging biomarker and clinical stage across age groups: (**A**) young patients with high Busyness stratified by stage (Low vs. High); (**B**) old patients with high Busyness stratified by stage (Low vs. High); (**C**) young patients with high stage stratified by Busyness (Low vs. High); (**D**) old patients with high stage stratified by Busyness (Low vs. High).

Figure 9 and Figure 10 evaluate whether the prognostic contrast between clinical stage (S) and the radiomic texture biomarker Busyness (B) persists after addressing class imbalance using Synthetic Minority Over-sampling Technique (SMOTE). Specifically, survival is examined in age-defined cohorts (young vs. old) to test two complementary questions: (i) Does stage meaningfully separate outcomes once Busyness-defined risk strata are fixed? and (ii) Does Busyness retain strong discriminatory power when evaluated within stage-defined cohorts, even under balanced subgroup composition?

#### 4.8.1. Stage-Based Survival Analysis Within Low-Busyness and Age Groups

Figure 9A,B assesses survival differences between High-Stage (High.S) and Low-Stage (Low.S) patients within the Low-Busyness (Low.B) group, stratified by age. Panel (A) focuses on young patients, where the survival curves for High.S and Low.S groups largely overlap, yielding a non-significant *p*-value of 0.48. This suggests that stage alone fails to effectively differentiate survival outcomes in younger individuals, even with SMOTE-Balanced Data. Panel (B) examines older patients, showing slightly improved separation between High.S and Low.S groups. However, the distinction remains weak, with a borderline *p*-value of 0.044, indicating only marginal survival stratification.

#### 4.8.2. Busyness-Based Survival Analysis Within Low Stage and Age Groups

A dramatically different outcome emerges when comparing Busyness (High.B vs. Low.B) within Low-Stage (Low.S) patients, stratified by age (Figure 9C,D). Panel (C) investigates young patients, where those with High Busyness experience markedly worse survival outcomes than those with Low Busyness. The separation is statistically significant, with a highly significant *p*-value (<0.0001), confirming Busyness as a powerful prognostic marker in younger individuals. Panel (D) evaluates older patients, showing an even more pronounced survival divergence between High.B and Low.B groups. The survival curves are clearly separated, with another highly significant *p*-value (<0.0001), reinforcing Busyness as a superior predictor of survival in older patients as well.

Taken together, these analyses demonstrate an apparent asymmetry in the prognostic contributions of stage and Busyness, even after correcting for class imbalance using SMOTE. When the stage is evaluated within Busyness-defined subgroups, survival separation remains weak and inconsistent across age groups, indicating that conventional staging provides limited independent prognostic information once radiomic heterogeneity is considered. In contrast, when Busyness is evaluated within stage-defined cohorts, survival curves separate sharply and reproducibly for both younger and older patients, with highly significant differences observed across Busyness levels. This pattern underscores Busyness as a dominant driver of survival stratification, capturing biologically relevant risk information not reflected in stage, and supports prioritizing radiomic texture biomarkers as primary stratifiers for survival prediction, with clinical stage serving a secondary, context-dependent role.

#### 4.8.3. Stage-Based Survival Analysis Within High-Busyness and Age Groups

Figure 9A,B assess whether stage (High.S vs. Low.S) effectively differentiates survival outcomes in patients with High Busyness, stratified by age. Panel (A) (Young Patients): The survival curves for High.S and Low.S groups show considerable overlap, with a non-significant *p*-value of 0.47. This indicates that stage alone does not provide meaningful survival stratification in young high-risk patients, even after balancing data with SMOTE. Panel (B) (Older Patients): The separation between High.S and Low.S groups slightly improves, but remains weak, with a *p*-value of 0.064, still above the threshold for statistical significance.

#### 4.8.4. Busyness-Based Survival Analysis Within High-Stage and Age Groups

A strikingly different trend emerges when comparing Busyness (High.B vs. Low.B) within High-Stage patients, stratified by age (Figure 10C,D). Panel (C), Young Patients: Among young individuals, those with High Busyness exhibit significantly worse survival outcomes than those with Low Busyness. The *p*-value (<0.0001) confirms the strong predictive power of Busyness, with a clear and consistent separation of survival curves. Panel (D), Old Patients: The survival disparity between High.B and Low.B is even more pronounced. Older patients with High Busyness experience a sharp decline in survival, reinforcing the robust prognostic value of this biomarker. The *p*-value (<0.0001) remains highly significant, demonstrating Busyness as a key determinant of survival risk. Taken together, these findings demonstrate a clear asymmetry in the prognostic utility of stage and Busyness within high-risk disease contexts. When stage is evaluated within High-Busyness subgroups, survival separation remains weak and statistically non-significant across both young and older patients, indicating that conventional staging provides limited incremental prognostic information once radiomic heterogeneity is accounted for, even after class imbalance correction. In contrast, when Busyness is evaluated within High-Stage cohorts, survival curves separate sharply and consistently for both age groups, with highly significant differences observed. This pattern confirms that Busyness captures dominant prognostic information that is not adequately reflected by clinical stage and remains robust under SMOTE-balanced conditions. Collectively, these results reinforce Busyness as a primary driver of survival stratification, while stage assumes a secondary, context-dependent role that becomes informative only after radiomic risk is considered.

### 4.9. Key Insights and Clinical Implications

These results further establish Busyness as a superior predictor of survival, even among high-stage patients, and emphasize key takeaways:

- Stage alone remains insufficient for effective survival stratification, even when applied within high-risk radiomic groups.
- Busyness offers significantly clearer separation of survival curves, underscoring its strong prognostic value in both young and old patients.
- SMOTE balancing corrects class imbalance, yet stage still fails to provide meaningful differentiation, whereas Busyness consistently enables precise risk identification.

This analysis reinforces the need to integrate radiomic biomarkers like Busyness into lung cancer survival modeling, as they offer superior predictive strength over traditional clinical variables like stage.

### 4.10. SMOTE-Balanced Survival Stratification: Busyness–Stage Interaction Across Gender Subgroups

**Figure 11:**
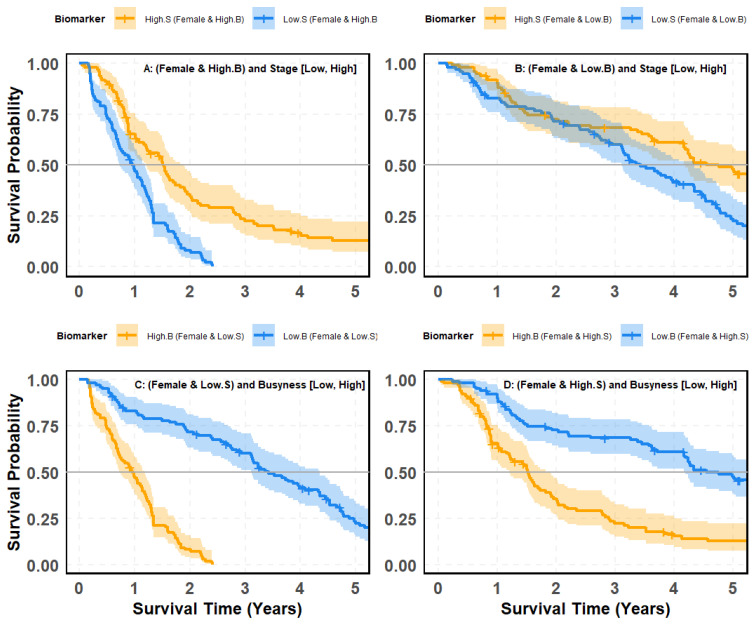
Kaplan–Meier survival curves based on SMOTE-balanced data illustrating patient risk groups from the interaction between the Busyness imaging biomarker and clinical stage in female patients: (**A**) females with high Busyness stratified by stage (Low vs. High); (**B**) females with low Busyness stratified by stage (Low vs. High); (**C**) females with low stage stratified by Busyness (Low vs. High); (**D**) females with high stage stratified by Busyness (Low vs. High).

**Figure 12:**
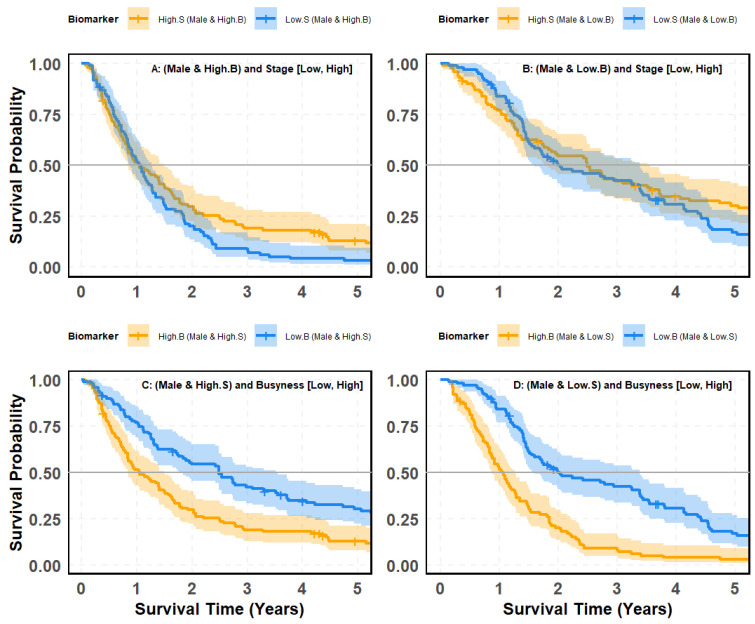
Kaplan–Meier survival curves based on SMOTE-balanced data illustrating patient risk groups from the interaction between the Busyness imaging biomarker and clinical stage in male patients: (**A**) males with high Busyness stratified by stage (Low vs. High); (**B**) males with low Busyness stratified by stage (Low vs. High); (**C**) males with high stage stratified by Busyness (Low vs. High); (**D**) males with low stage stratified by Busyness (Low vs. High).

Figure 11 and Figure 12 evaluate whether the prognostic contrast between clinical stage (S) and the radiomic texture biomarker Busyness (B) persists after correcting for class imbalance using Synthetic Minority Over-sampling Technique (SMOTE), with survival outcomes examined across gender-defined cohorts (female vs. male). This analysis addresses two complementary questions: (i) whether tumor stage meaningfully differentiates survival once Busyness-defined risk strata are fixed within each gender, and (ii) whether Busyness retains strong discriminatory power when evaluated within stage-defined cohorts, even under balanced subgroup composition by systematically comparing stage- and Busyness-based stratification across male and female patients. These analyses assess whether observed survival differences reflect independent prognostic contributions from gender or are primarily driven by radiomic heterogeneity captured by Busyness.

#### 4.10.1. Survival Stratification in Female Lung Cancer Patients: Comparing Stage and Busyness

Figure 11 evaluates survival outcomes for female patients, analyzing combinations of stage (High vs. Low) and Busyness (High vs. Low) to determine which factor more effectively stratifies survival risk. Panels (A) and (B) assess stage as a predictor of survival within groups already stratified by Busyness. Panel (A) (High-Busyness Group): While the *p*-value is statistically significant (<0.0001), the visual separation between High-Stage and Low-Stage survival curves is modest. Confidence intervals overlap considerably, indicating limited survival discrimination in females with High Busyness. Panel (B) (Low-Busyness Group): Among females with Low Busyness, survival curves remain closely aligned, with only a moderate *p*-value (0.0021). Despite statistical significance, the stage-based separation is weak, suggesting that stage alone is not a reliable predictor, even when Busyness is controlled. A significantly stronger stratification emerges when evaluating Busyness within stage groups. Panel (C) (Low-Stage Group): Among Low-Stage females, those with High Busyness have dramatically lower survival probabilities than those with Low Busyness. The separation between curves is visually clear, with a highly significant *p*-value (<0.0001). Panel (D) (High-Stage Group): A similar but even more pronounced pattern appears in High-Stage females, where High Busyness is strongly associated with worse survival outcomes. The survival curves show distinct separation, confirmed by another highly significant *p*-value (<0.0001).

#### 4.10.2. Survival Stratification in Male Lung Cancer Patients: Comparing Stage and Busyness

Figure 12 examines survival outcomes in male lung cancer patients, focusing on the relative predictive power of stage (High vs. Low) and Busyness (High vs. Low) to determine the most effective stratification method. Panels (A) and (B) assess stage as a predictor of survival within Busyness-defined subgroups. Panel (A), High-Busyness Group: Among males with High Busyness, survival outcomes differ between High Stage and Low Stage, but the curves exhibit considerable overlap. While the *p*-value (0.035) is statistically significant, the actual separation is limited, reducing the practical utility of stage in this context. Panel (B), Low-Busyness Group: In males with Low Busyness, survival curves for High and Low Stage remain moderately overlapping, and the *p*-value (0.06) suggests borderline significance. This reinforces that stage alone provides weak stratification, even when patients are pre-grouped by Busyness. A much stronger separation appears when evaluating Busyness within Stage-defined groups. Panel (C), High-Stage Group: Among High-Stage males, those with High Busyness experience significantly worse survival than those with Low Busyness. The survival curves are clearly and consistently separated, with a highly significant *p*-value (<0.0001), underscoring Busyness as a powerful prognostic factor. Panel (D), Low-Stage Group: The impact of Busyness is even more dramatic in Low-Stage males. Patients with High Busyness show a steep drop in survival probabilities, while those with Low Busyness maintain substantially better survival. The *p*-value (<0.0001) confirms the strong predictive strength of Busyness.

## 5. Discussion

The findings of this study underscore the superiority of radiomic biomarkers, particularly Busyness, over conventional clinical variables such as stage, age, and gender in predicting lung cancer survival outcomes. Our survival analyses consistently demonstrate that Busyness provides significantly better stratification, with highly distinct survival curves and minimal overlap in confidence intervals. This suggests that tumor texture complexity, as quantified by radiomic analysis, encapsulates critical prognostic information not captured by traditional clinical parameters.

The stratified analyses further reinforce these conclusions. When patients were grouped by age, survival probabilities remained significantly different between high and low Busyness subgroups, with older patients showing worse survival outcomes. However, the predictive power of Busyness remained strong in both young and old cohorts, showing its broad applicability. Similarly, within gender-defined groups, females generally showed better survival outcomes than males, but the Busyness-based classification demonstrated far clearer separation, reinforcing its independent prognostic value. These findings align with emerging evidence suggesting that tumor heterogeneity, as captured by imaging features, plays a pivotal role in disease progression and treatment response.

The clinical relevance of these findings should also be interpreted in the context of the post-COVID-19 transformation of thoracic imaging practice. During the COVID-19 pandemic, chest computed tomography became one of the most widely acquired imaging modalities worldwide, accelerating clinical adoption of AI-assisted image analysis and establishing the technical and regulatory infrastructure for large-scale imaging analytics. This period also entrenched the paradigm of opportunistic lung cancer screening, whereby CT scans obtained for non-oncologic indications are systematically reviewed for incidental pulmonary nodules. As emphasized in the recent healthcare data analytics literature, this shift has moved the clinical challenge beyond detection toward robust downstream risk stratification once malignancy is identified. Within this landscape, the strong prognostic performance of Busyness highlights its potential role in addressing an emerging post-COVID clinical need by providing a quantitative and clinically actionable measure of tumor aggressiveness [34,35]. In this setting, radiomic biomarkers capable of capturing tumor aggressiveness may inform not only prognostic assessment but also downstream clinical decisions regarding surveillance intensity and therapeutic escalation. While recent studies have emphasized detection efficiency, screening utilization, and large-scale imaging analytics in the COVID-19 era, comparatively little attention has been directed toward quantitative prognostic characterization following detection, a gap that the present study directly addresses.

Taken together, these findings position the present study within what can be considered a second wave of artificial intelligence applications in thoracic oncology. Whereas the first wave—accelerated during the COVID-19 pandemic—primarily emphasized detection, segmentation, and workflow efficiency in chest CT imaging, the current clinical challenge has shifted toward prognostic characterization once malignancy is identified. By demonstrating that a radiomic texture feature can robustly outperform conventional clinical variables in survival stratification, even under class-imbalanced conditions, this work advances AI utility beyond detection toward clinically actionable risk assessment. In this context, Busyness provides a quantitative surrogate of tumor aggressiveness that may inform surveillance intensity and treatment decision-making in post-COVID, high-throughput imaging environments.

### 5.1. Use of Radiomics and Machine Learning in Lung Cancer Stratification

This study explores the integration of biomarkers (radiomics) and machine learning (ML) to improve lung cancer stratification. Radiomics are imaging biomarkers obtained using advanced medical imaging techniques that extract quantitative features from medical images (such as CT or MRI scans) to capture tumor characteristics that may not be visible to the human eye [13,36,37,38,39]. Machine learning models are employed to analyze these features and stratify patients into appropriate lung cancer groups.

### 5.2. Key Findings and Implications

These results confirm that while age is an important factor in survival prediction, Busyness exhibits even stronger stratification power, particularly within age-defined subgroups. The more pronounced separation in survival curves underscores the superior discriminatory ability of radiomic biomarkers such as Busyness. This analysis further supports the integration of radiomic features into survival modeling to improve risk assessment and enhance patient stratification in lung cancer prognosis. Notably, the age-based findings demonstrate that Busyness provides far stronger survival stratification within gender groups than gender does within Busyness-defined subgroups, reinforcing that radiomic biomarkers offer superior prognostic value compared with traditional demographic factors such as sex. These findings underscore the importance of incorporating radiomic features into survival modeling, as they enable enhanced patient stratification and more precise risk assessment in lung cancer prognosis. The robustness of radiomic biomarkers such as Busyness is further evident when class imbalance is addressed using SMOTE. Even under balanced subgroup conditions, tumor stage remains a weak predictor of survival, offering limited separation of survival curves. In contrast, Busyness consistently outperforms stage and provides significant survival stratification in both younger and older patients. Collectively, these observations suggest that multimodal integration yields more precise and clinically meaningful insights, underscoring the importance of incorporating quantitative imaging biomarkers into prognostic frameworks, rather than relying solely on traditional clinical staging.

### 5.3. Background and Prior Work

Previous studies have shown that radiomic features are associated with outcomes in several cancer types, including lung cancer. Texture-, shape-, and intensity-based features have been linked to survival, treatment response, and metastatic risk in multiple studies [13,36,37,38,39]. Many lung cancer radiomics studies have focused on classification problems, such as identifying tumor subtypes or predicting stages, or on enhancing model performance. Fewer studies have examined the prognostic value of individual radiomic features using survival-based analyses and direct comparison with standard clinical variables. In this context, the present study differs from prior work in several important ways. First, rather than introducing a novel classification model, we adopt a survival-based evaluation framework that prioritizes time-to-event outcomes, which are more clinically relevant for prognosis. Second, we emphasize biomarker stability and reproducibility through repeated Elastic Net-based Frequency Ranking across multiple regularization settings, rather than relying on single-model feature selection. Third, we explicitly benchmark radiomic biomarkers against conventional clinical predictors, including tumor stage, age, and sex, demonstrating that texture-based imaging features—particularly Busyness—provide substantially stronger prognostic discrimination. These distinctions position the current work as a complementary and clinically focused contribution to the existing literature on lung cancer radiomics.

From a biological perspective, the radiomic feature *Busyness* reflects rapid spatial variations in voxel intensity within the tumor and is derived from the Neighborhood Gray-Tone Difference Matrix (NGTDM). High Busyness values indicate pronounced local heterogeneity, suggesting frequent and abrupt changes in tissue density across small spatial scales. Such imaging patterns may correspond to underlying histopathological characteristics associated with aggressive tumor behavior, including heterogeneous cellular density, irregular stromal organization, necrosis, hypoxia, and variations in tumor vascularization. These microenvironmental features are known to influence tumor progression, treatment resistance, and metastatic potential.

While radiomic features do not directly map to specific histological structures, Busyness may serve as a noninvasive surrogate marker of tumor heterogeneity, capturing phenotypic complexity that is not fully represented by conventional clinical variables such as tumor stage. The strong and consistent prognostic performance of Busyness observed in this study supports the notion that texture-based radiomic features encode biologically meaningful information related to tumor aggressiveness. Future work integrating radiomic biomarkers with histopathological, molecular, and genomic data will be essential to elucidate the biological underpinnings of Busyness further and to strengthen its interpretability and clinical utility.

Radiomic biomarkers such as Busyness may also play a complementary role alongside molecular and genomic markers in lung cancer prognostication. While genomic alterations capture specific oncogenic drivers and pathway-level information, radiomic features provide a noninvasive, whole-tumor assessment of spatial heterogeneity that reflects phenotypic expression of underlying biology. Integrating radiomic features with molecular data, such as mutation status, gene expression profiles, or immune-related markers, has the potential to improve risk stratification by linking tumor genotype with macroscopic imaging phenotypes. Such multimodal approaches could enable more comprehensive prognostic models that combine biological specificity with spatial and phenotypic context. Future studies incorporating joint radiomic–genomic analyses and prospective validation will be essential to fully realize the clinical potential of these integrated precision oncology frameworks.

### 5.4. Model Performance and Over-Sampling Strategies

The study evaluated over-sampling strategies to explore the impact of class imbalance on subgroup-based stratification analyses. SMOTE was applied in an exploratory manner to assess the stability of observed survival separation patterns under more balanced subgroup conditions. Importantly, the primary survival analyses and conclusions regarding prognostic performance were derived from the original, non-oversampled dataset. Across both original and SMOTE-balanced analyses, Busyness consistently demonstrated strong discriminatory ability, supporting its robustness as a radiomic biomarker. In contrast, tumor stage alone showed limited prognostic value, even within high-risk subgroups. While stage remains an essential clinical metric, these findings align with prior evidence suggesting that conventional staging may not fully capture tumor biological heterogeneity. Integrating radiomic biomarkers with staging information may therefore enhance risk-adapted prognostic modeling.

The use of SMOTE represents an important methodological limitation. While over-sampling techniques are effective in classification settings, they may alter the underlying time-to-event structure in survival data. In this study, SMOTE was not used to derive primary survival inferences but rather to explore the robustness of stratification patterns in highly imbalanced subgroups. Future work will focus on survival-specific imbalance handling methods and validation in larger, multi-institutional datasets.

### 5.5. Limitations and Future Directions

Despite the promising findings reported in this study, several limitations warrant consideration. First, all analyses were conducted using a single retrospective dataset, which may limit the representativeness of the cohort and the generalizability of the results to broader patient populations, imaging protocols, and clinical settings. We attempted to limit overfitting by using repeated cross-validation and Elastic Net regularization. However, the number of radiomic features remains high compared with the sample size. This is a common issue in radiomics studies and cannot be fully avoided. As a result, model performance may be overestimated, even when regularization is used.

Furthermore, class imbalance was present in several subgroup analyses. SMOTE was used only to check whether survival trends were preserved when subgroup sizes were more balanced. Because over-sampling can interfere with survival time information, SMOTE was not used for primary analysis. All main findings were obtained from the original dataset without over-sampling. Future studies should focus on survival-specific approaches for handling imbalance and on external validation using larger, multi-institutional datasets. Finally, while Busyness was selected as a representative radiomic biomarker based on its stability and robustness in feature selection, integrating additional radiomic features alongside molecular and genomic biomarkers has the potential to strengthen prognostic models further and support the development of more comprehensive, risk-adapted treatment strategies.

## 6. Conclusions

This study highlights the critical role of radiomic biomarkers in enhancing lung cancer survival prediction, demonstrating that Busyness outperforms traditional clinical variables such as stage, age, and gender. The findings suggest that tumor texture complexity, as captured through radiomics, offers a more precise and clinically actionable approach to risk stratification. Our results further validate the robustness of radiomic features, particularly when coupled with machine learning techniques like SMOTE to address class imbalance.

Stage has conventionally been a key factor in survival outcomes, with higher stages associated with shorter survival times. Age plays a role in survival, but does not show noticeable differences across groups. Gender does not show a strong survival impact, as male and female distributions largely overlap. Violin plots reinforce that survival time varies more within lower-stage patients, likely due to treatment responses or other prognostic factors. The presence of outliers, showing some individuals experience much longer survival despite high-risk factors. Busyness provides far superior survival stratification compared to stage, with much clearer separation of survival curves. Stage alone is insufficient for meaningful survival discrimination, even when controlling for Busyness. The group “Low Stage and Low Busyness” consistently demonstrates the highest survival probabilities, supporting the idea that combining radiomic biomarkers with clinical variables enhances survival modeling. Busyness is a more informative and clinically actionable variable than stage, demonstrating far stronger visual separation. Busyness significantly outperforms stage in stratifying male lung cancer survival outcomes. Stage alone offers weak survival discrimination, even within Busyness-defined subgroups. The combination “Low Stage and Low Busyness” consistently associates with the best survival probabilities, highlighting the protective nature of low-risk radiomic and clinical features. Busyness provides greater prognostic insight into patient survival than tumor stage. Incorporating radiomic imaging biomarkers such as Busyness into survival models enables more precise and clinically meaningful risk stratification in male patients with lung cancer. These findings establish Busyness as a key prognostic biomarker, reinforcing its predictive utility in lung cancer survival modeling, particularly when paired with demographic factors and advanced data-balancing techniques. These findings further demonstrate that, particularly among female lung cancer patients, radiomic imaging biomarkers such as Busyness significantly outperform tumor stage in survival prediction, providing a more precise and reliable approach to patient stratification. In male lung cancer patients, the inclusion of the radiomic imaging biomarker Busyness enhances the precision and clinical relevance of survival-based risk stratification. Given the limitations of stage-based prognostication, integrating radiomic biomarkers into clinical workflows could significantly refine treatment decision-making, ultimately improving patient outcomes. Future research should focus on expanding the scope of radiomic analysis, incorporating genetic and molecular markers, and validating findings across diverse populations to fully realize the potential of radiomics in precision oncology.

## Figures and Tables

**Table 1:** Baseline demographic and clinical characteristics of the NSCLC cohort.

Characteristic	Value
Number of patients (N)	398
Age, mean ± SD (years)	68.1 ± 10.1
Age, median [IQR] (years)	68.7 (61.3–75.9)
Gender: Male, *n* (%)	273 (68.6%)
Gender: Female, *n* (%)	125 (31.4%)
Age group: Young (≤median), *n* (%)	199 (50.0%)
Age group: Old (>median), *n* (%)	199 (50.0%)
Stage I, *n* (%)	84 (21.1%)
Stage II, *n* (%)	37 (9.3%)
Stage III (IIIa + IIIb), *n* (%)	277 (69.6%)
Low Biomarker (Busyness), *n* (%)	199 (50.0%)
High Biomarker (Busyness), *n* (%)	199 (50.0%)
Survival time, median (range) (years)	1.50 (0.03–11.85)
Event status: Death, *n* (%)	351 (88.2%)
Event status: Censored, *n* (%)	47 (11.8%)

**Table 2:** Top-16 Biomarkers identified by Elastic Net Frequency Ranking.

Rank	Biomarker/Radiomic	Frequency Rank	Group Name
1	Busyness	1053	Neighborhood Gray-Tone Difference Matrix (NGTDM)
2	Maximum 2D Diameter Slice	1053	Shape/Morphological Features
3	Size Zone Non-Uniformity	1053	Gray-Level Size Zone Matrix (GLSZM)
4	Strength	1053	Neighborhood Gray-Tone Difference Matrix (NGTDM)
5	Maximum 2D Diameter Row	1053	Shape/Morphological Features
6	Surface Area	701	Shape/Morphological Features
7	MajorAxisLength	552	Shape/Morphological Features
8	LowGrayLevelZoneEmphasis	361	Gray-Level Size Zone Matrix (GLSZM)
9	SurfaceVolumeRatio	297	Shape/Morphological Features
10	Dependence Non-Uniformity	200	Gray-Level Difference Method (GLDM)
11	Gray-Level Non-Uniformity.2	200	Gray-Level Size Zone Matrix (GLSZM)
12	Least Axis Length	200	Shape/Morphological Features
13	Maximum 3D Diameter	200	Shape/Morphological Features
14	Mesh Volume	200	Shape/Morphological Features
15	Run Length Non-Uniformity	200	Gray-Level Run Length Matrix (GLRLM)
16	Voxel Volume	200	Shape/Morphological Features

## Data Availability

Data is publicly available and cited in the manuscript. Data Cite: NSCLC-RADIOMICS: The Cancer Imaging Archive (TCIA). Available online: https://www.cancerimagingarchive.net/collection/nsclc-radiomics/ (accessed on 23 June 2025).

## Abbreviations

NSCLC	Non-Small-Cell Lung Cancer
SCLC	Small-Cell Lung Cancer
TNM	Tumor–Node–Metastasis
ML	Machine Learning
AI	Artificial Intelligence
LASSO	Least Absolute Shrinkage and Selection Operator
CT	Computed Tomography
Cox PH	Cox Proportional Hazards
KM	Kaplan–Meier
GLDM	Gray-Level Dependence Matrix
GLRLM	Gray-Level Run Length Matrix
GLSZM	Gray-Level Size Zone Matrix
IBSI	Image Biomarker Standardization Initiative
NGTDM	Neighborhood Gray-Tone Difference Matrix
RSS	Representativeness Strength Score
SMOTE	Synthetic Minority Over-Sampling Technique
TCIA	The Cancer Imaging Archive
